# Analysis of Sanitizer Rotation on the Susceptibility, Biofilm Forming Ability and Caco-2 Cell Adhesion and Invasion of *Listeria*

**DOI:** 10.3390/pathogens11090961

**Published:** 2022-08-24

**Authors:** Md Asfakur Rahman, Nirakar Sahoo, Veerachandra Yemmireddy

**Affiliations:** 1 Department of Biology, University of Texas Rio Grande Valley, Edinburg, TX 78539, USA; 2 School of Earth, Environmental and Marine Sciences, University of Texas Rio Grande Valley, Edinburg, TX 78539, USA

**Keywords:** *Listeria monocytogenes*, resistance, repeated exposure, biofilm, pathogenicity, sanitizers

## Abstract

The purpose of this study was to determine the effect of sanitizer use conditions on the susceptibility, biofilm forming ability and pathogenicity of *Listeria* *monocytogenes*. Two different strains of *L. monocytogenes* and a non-pathogenic *L. innocua* were exposed to sodium hypochlorite, benzalkonium chloride and peroxyacetic acid at different concentrations (4 to 512 ppm) and treatment times (30 s to 5 min), respectively. Under the tested conditions, no significant difference (*p >* 0.05) in reduction was observed among the three tested sanitizers. A reduction of 1 to 8 log CFU/mL was observed depending upon the sanitizer concentration and treatment times. The survived cells at the highest sublethal concentration and treatment time of a particular sanitizer upon re-exposure to the same or different sanitizer showed either no change or increased susceptibility when compared to parent strains. Upon repeated exposure to sanitizers at progressively increasing concentrations from 1 to 128 ppm, *L. innocua* was able to survive concentrations of up to 32 ppm benzalkonium chloride and 64 ppm peroxyacetic acid treatments, respectively. At the tested sub-lethal concentrations, no significant difference (*p >* 0.05) in biofilm formation was observed among the tested strains. Caco-2 interaction with *L. innocua* showed a reduction in invasion ability with sublethal concentrations of sanitizers.

## 1. Introduction

*Listeria monocytogenes* is a major foodborne pathogen of concern with several reported outbreaks in recent times. It causes listeriosis in susceptible individuals with a case fatality rate of 20 to 30% [1] and persists well in the environment [2]. One of the main contamination routes for *L. monocytogenes* is through cross-contamination from equipment/machines to food during processing [3]. Studies have revealed that certain strains of *L. monocytogenes* can become well established in a food processing facility in locations such as floor drains and remain members of the resident microbial flora for months or years [4]. The ability of *L. monocytogenes* to adhere to surfaces and form biofilms has been demonstrated in different studies [5]. Furthermore, *Listeria* can adhere to surfaces and form biofilms that can provide protection from the action of sanitizers and help to persist in food processing environments for extended periods [6]. One consequence of biofilm formation is the acquisition of (adaptive) resistance to cleaning and disinfection agents, which can lead to serious economic and health problems [7,8]. Some strains can adapt to hostile environments, developing mechanisms of resistance, and persist in food processing plants for several years [6]. 

Industrial disinfectants including quaternary ammonium compounds, alcohols, chlorinated compounds, and other oxidizing agents such as peracetic acid, ozone and peroxide derivatives among others, are commonly used [7,9,10]. Peroxyacetic acid (PAA) is an environment-friendly sanitizer that decomposes and produces no harmful by-product [11]. PAA, depending on requirements, can be used at concentrations from 100–200 ppm [12]. Chlorine-based disinfectants, such as sodium hypochlorite, are oxidizing compounds widely used in the food industry due to their broad-spectrum bactericidal activities, high efficacy, and low cost [13]. Quaternary ammonium compounds (QAC), such as benzalkonium chloride, are cationic surfactants that act through the disruption of lipid membrane bilayers, being effective against several photogenic microorganisms, especially Gram-positive bacteria [14]. QACs are usually applied at concentrations of 200 ppm [12]. However, sublethal sanitizer concentrations may lead to the risk of developing tolerance and/or resistance to the same sanitizers [15] or to different sanitizers and antimicrobial agents [16]. The potential resistance or tolerance could be attributed to physiological changes in outer cell membrane phospholipid composition, or it might be related to specific genotypic changes [17]. Studies have reported that higher biofilm populations are enumerated on the persistent or more tolerant strains [18], whereas other studies found no association between persistence and these specific phenotypic characteristics [19].

There exists a growing debate on the rotation of sanitizers to help break the cycle of tolerance or resistance development in *Listeria*, but sufficient scientific evidence to fully support or refute the benefits of sanitizer rotation is lacking. Bland et al. [20] provided a comprehensive review on this topic. Moreover, the effect on the survivability and biofilm forming ability of *L. monocytogenes* when subjected to sublethal doses of sanitizers needs better understanding. Furthermore, the pathogenesis of *L. monocytogenes* starts upon ingestion through contaminated food, where it survives exposure to high acidity, bile salts, non-specific inflammatory attacks, and proteolytic enzymes from the host system [21]. After surviving this stage, *L. monocytogenes* adheres to and enters both phagocytic and non-phagocytic cells of the host through the assistance of surface proteins called internalins [22]. The infection process of the host cells by *L. monocytogenes* involves several distinct stages: adhesion and invasion of host cells, realization by host cells, and lysis of vacuole, intracellular multiplication, and intercellular spreading to adjacent cells [23]. As per our knowledge, it is still unclear how sublethal exposure to sanitizers will impact intestinal cell adhesion and the invasion of *Listeria.* Any study focusing on the above-mentioned aspects of *Listeria* will help to elucidate the effects of lethal and sublethal exposures to the same or different sanitizers on its susceptibility, biofilm forming ability and pathogenicity. Thus, the main objectives of this study were (i) to determine the in vitro susceptibility of *L. monocytogenes* and *L. innocua* when subjected to different sanitizers and their use conditions; (ii) to determine the effects of re-exposure and repeated exposure to the same or different sanitizers on the susceptibility of *L. monocytogenes* and *L. innocua*; (iii) to determine the effect of sublethal sanitizer concentrations on the biofilm forming ability of *L. monocytogenes* and *L. innocua*; and (iv) to understand the cell adhesion and invasion potential of *L. innocua* before and after exposure to sublethal concentrations of select sanitizers.

## 2. Materials and Methods

### 2.1. Selection of Bacterial Strains and Inoculum Preparation

Two different serotypes of *Listeria monocytogenes*, namely *L. monocytogenes* 101M (serotype 4b, beef associated outbreak isolate) and *L. monocytogenes* F8385 (serotype 1/2b, carrot associated outbreak isolate), and a non-pathogenic surrogate, *L. innocua* (ATCC 51742, plant derived cabbage isolate), were tested in this study to determine the strain/serotype variability. All the tested strains were stored at −80 °C in tryptic soy broth (TSB; Hardy diagnostic™, Santa Maria, CA, USA) containing 25% glycerol. Prior to each experiment, the frozen cultures were activated by two successive passages by first inoculating 100 µL in 10 mL TSB with 0.6% yeast extract (TSB-YE) and incubating at 37 °C for 18–20 h. Following incubation, the cells were harvested by centrifugation (Model 5920R, Eppendorf™, Hamburg, Germany) at 4000× *g* for 10 min at 4 °C. The resultant supernatant was decanted, and the pellet was re-suspended in 10 mL of sterile phosphate-buffered saline (PBS, pH 7.2). This procedure was repeated twice, and the final pellets of individual strains were prepared in PBS to achieve a cell concentration of 10^7–8^ CFU/mL. Cell concentration was adjusted by measuring the absorbance at 600 nm using a UV/Vis spectrophotometer (Bio spectrometer, Eppendorf™, Germany) and confirmed by plating 100 µL portions of appropriate serial dilutions on tryptic soy agar (TSA) plates and incubating at 37 °C for 24–48 h. 

### 2.2. Preparation of Sanitizer Solutions

Three types of sanitizers: (i) sodium hypochlorite (SHC) (5% available chlorine, Ricca Chemical Company, Arlington, TX, USA), (ii) benzalkonium chloride (BAC) (17% *W*/*V* solution, Spectrum Chemical, Gardena, CA, USA), and (iii) peroxyacetic acid (PAA) (SaniDate^®^ 15, Biosafe Systems, East Hartford, CT, USA) were used in this study. Chlorine stock solutions were prepared using sodium hypochlorite with hydrochloric acid as an acidulant to adjust the pH to 6.5, while BAC and PAA stock solutions were prepared by diluting with PBS and without pH adjustment. The concentrations of all sanitizers were adjusted to 4, 8, 16, 32, 64, 128, 256, and 512 ppm. Free chlorine concentration was determined with a colorimetric method by following DPD-free chlorine assays using a DR900 portable colorimeter (HACH^®^, Loveland, CO, USA). The corresponding concentrations of BAC and PAA were determined by using QAC Multi Quat™ (Bartovation, New York, NY, USA) and MQuant^®^ (MilliporeSigma, Burlington, MA, USA) test strips, respectively. 

### 2.3. Single Exposure, Re-Exposure, and Repeated Exposure Treatments

The susceptibility of *L. innocua* and *L. monocytogenes* strains to different sanitizer treatments was determined based on the Clinical and Laboratory Standards Institute (CLSI) microbroth dilution method [24] with modifications by following Riazi et al. [25] An aliquot of 100 μL of individual inoculum was added separately into 900 μL of each sanitizer at concentrations 4, 8, 16, 32, 64, 128, 256, and 512 ppm in a 96-well plate. The contents were thoroughly mixed by aspirating twice with a multichannel micropipette and subjected to 0.5, 1-, 2.5- and 5-min treatments, respectively. Control samples with just PBS in place of sanitizer were also included. After the treatment, 100 μL of the sample were collected and added into 900 μL D/E neutralizing broth, and the microplate was held at 22 °C for 10 min. Serial dilutions were prepared in PBS, and the appropriate dilutions were plated on TSA plates and incubated at 37 °C for 24–48 h. The log reductions were calculated as CFU/mL. The cells that survived the highest sanitizer concentration and treatment time (identified as 256 ppm; 30 s or 1 min) from the above procedure were isolated after the incubation period. These isolated colonies were regrown to a cell concentration of 10^7–8^ CFU/mL as described in Section 2.1 and again subjected to either the same sanitizer at the same concentration (256 ppm) where they were originally isolated or to a different sanitizer at varying concentrations (64, 128, 256, 512 ppm), as described previously. The results were compared with one-time sanitizer exposed and unexposed cells for the similarities or differences in their susceptibility or tolerance levels. 

In the last stage, the survived cells from the above steps were repeatedly exposed to progressively increasing concentrations of BAC and PAA starting from 1 ppm and evaluated by following Riazi et al. [25] with modifications. Briefly, a 500 µL aliquot of an overnight grown inoculum was added into 4.5 mL of Mueller Hinton broth containing different concentrations (1, 4, 8, 16, and 32 ppm) of BAC or PAA and incubated at 37 °C for 24 h with shaking. Sodium hypochlorite was not included in these experiments due to its inability to maintain desired sanitizer concentrations in MH broth during the 24 h incubation periods. The following day, inoculum from test tubes showing turbidity were transferred to subsequently higher concentrations of sanitizer (i.e., BAC or PAA) containing fresh growth media and returned to the incubator. The tube(s) that did not show any turbidity were transferred to fresh media containing no sanitizer(s) to confirm the absence of any survivors upon plating the following day on TSA. This was further confirmed by qualitative color change determination based on a live or dead cell assay using a 0.5% 2, 3, 5-triphenyltetrazolium chloride (INT) indicator (Thermo Scientific™, Waltham, MA, USA). In this way, the concentrations of BAC and PAA under which *Listeria* survived upon repeated exposures were determined. 

### 2.4. Biofilm Production Assay

The biofilm production assay was performed by following the procedure described in Ammendolia et al. [26]. Briefly, the selected strains of *Listeria* in their original unexposed form (i.e., parent strains) and their corresponding one-time sanitizer exposed, re-exposed, and repeatedly sanitizer exposed cells were cultured overnight in TSB-YE. Followed by incubation, the cells were harvested and diluted in PBS to reach a concentration of 10^7−8^ CFU/mL. An aliquot of 100 µL of inoculum in TSB-YE (i.e., 100-fold dilution) was added to a 96-well plate containing 100 µL of sanitizer(s) of different concentrations (4, 8, 16, 32 ppm), thus leading to a net sanitizer concentration of 2, 4, 8, and 16 ppm in the respective wells, and incubated for 24, 48, or 72 h at 37 °C in a static condition. After respective incubation times, the wells of the plate were washed five times with sterile distilled water to remove non-attached cells and allowed to dry at 37 °C for 1 h. After that, the wells were stained with 1% crystal violet and left at room temperature for 30 min. Following five times washing again with sterile distilled water, the remaining crystal violet was eluted using 95% ethanol for 15 min. The absorbance reading of the collected biomass in the wells was determined at 590 nm using microplate reader (Bio-Rad™, Hercules, CA, USA).

### 2.5. Caco-2 Cell Interaction Assay

#### 2.5.1. Inoculum Preparation 

The cells exposed to sublethal concentrations (4, 16, and 32 ppm) of BAC and PAA during repeated exposure as described in Section 2.3 were saved in TSA slants for later use. These cells along with the parent strains were activated in TSB-YE following 24 h incubation at 37 °C. The cells were harvested by following the procedure described previously.

#### 2.5.2. Preparation of Mammalian Cell Culture

The human intestinal cell line “Caco-2 (ATCC#HTB-37)” was grown in Dulbecco’s modified Eagle’s medium (DMEM) with 10% fetal bovine serum at 37 °C and 5% CO_2_ in a humidified incubator.

#### 2.5.3. Adhesion and Invasion Assay

Adhesion and invasion potential of *L. innocua* on the Caco-2 cells was determined by following the method proposed by Reddy and Austin [27]. Briefly, Caco-2 cells were grown in 24-well plates until about 80% confluency was reached for both adhesion assay and invasion assay. For the adhesion assay, 20 µL of *L. innocua* inoculum in PBS at a concentration of 10^7^ CFU/mL was cultured with Caco-2 cells for 1 h at 37 °C in a 5% CO_2_ atmosphere. After 1 h incubation, the Caco-2 cells were washed 3 times with PBS to remove loosely attached bacteria. After washing, 300 µL of 0.1% Triton X-100 were used to lyse the cells, and the mixture was plated on TSA. For the invasion assay, the same volume of bacteria as above was added to Caco-2 cells and incubated for 2 h at 37 °C in a 5% CO_2_ atmosphere. The bacteria were then washed 3 times with PBS, followed by incubation with Gentamycin (50 mg/mL) in the fresh medium for 15 min at 37 °C to kill extracellular bacteria. Finally, the mixture was plated on TSA. The percentage of adhesion and invasion of *L. innocua* was calculated by normalizing the bacteria adhered to or invaded into Caco-2 cells to the total number of bacteria in the inoculum. The data were presented as fold change of *L. innocua* adhered or invaded when previously exposed to or not exposed to the action of sanitizers.

#### 2.5.4. Confocal Imaging of Invaded *L. innocua* in Caco-2 Cells

For confocal imaging, the Caco-2 cells were cultured on a glass coverslip in 24-well culture dishes. The same invasion experiment was carried out as described above. After the experiment, the Caco-2 cells were then fixed with 100% methanol for 10 min at 4 °C, followed by 3× wash with PBS, and then incubated with 5 µM SYTO-9 dye (Invitrogen™, Waltham, MA, USA) for 1 h at room temperature. After the incubation, the cells were washed thrice with PBS. The coverslips were mounted on a glass slide with Fluoromount-G and imaged with Olympus Fluoview FV10i confocal microscope (Olympus, Waltham, MA, USA) using a 60× objective. The images were further processed with ImageJ (NIH) software. 

### 2.6. Statistical Analysis

All experiments were conducted in triplicate. The data were analyzed by the analysis of variance (ANOVA) procedure using SPSS^TM^ (IBM^®^ Version 25, Armonk, NY, USA). Tukey’s post hoc test was used to determine the significant differences in mean values with significance considered at *p* < 0.05. 

## 3. Results and Discussion

### 3.1. Effect of Type of Sanitizer, Sanitizer Concentration, and Treatment Time on the Log Reduction 

**Figure 1:**
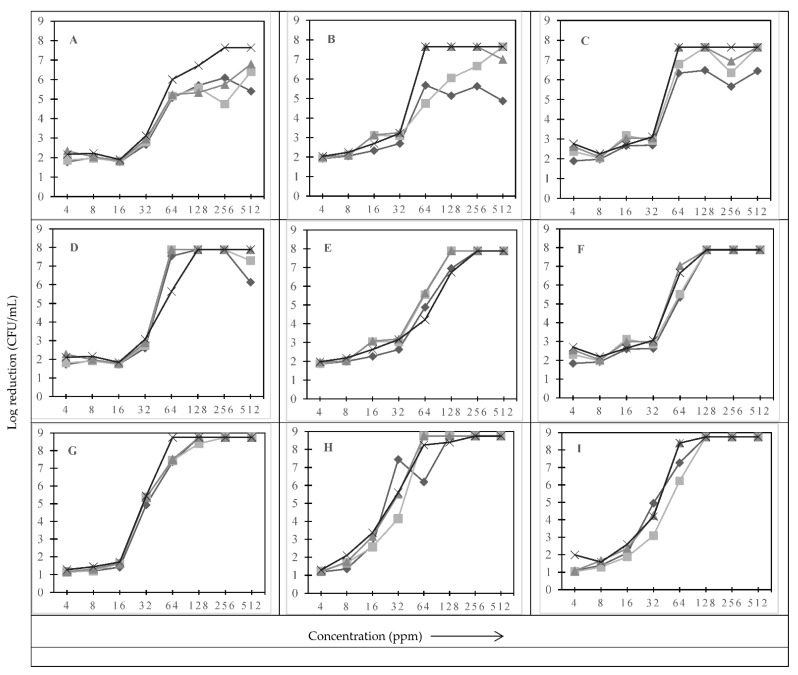
Effect of type of strain, type of sanitizer and its concentration, and treatment time on the log reduction. (**A**–**C**) Log reduction of *L. innocua* subjected to different concentrations of sodium hypochlorite (SHC), peroxyacetic acid (PAA), and benzalkonium chloride (BAC), respectively. (**D**–**F**) The same sanitizer treatments against *L. monocytogenes* (101M, *Lm-1*), respectively. (**G**–**I**) The same sanitizer treatments against *L. monocytogenes* (F8385, *Lm-2*), respectively. 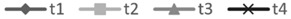 represent treatment times of 30 s, 1, 2.5, and 5 min, respectively.

Figure 1 shows the effect of type of sanitizer, concentration of sanitizer and treatment time on the log reduction of *L. monocytogenes* and *L. innocua.* A reduction of 1.78 to 6.05 log CFU/mL was observed when *L. innocua* was subjected to sodium hypochlorite (SHC) treatment at 4 to 512 ppm for 30 s (Figure 1A). As expected, increasing the sanitizer concentration from 4 to 512 ppm increased the log reduction. However, no significant difference (*p >* 0.05) in log reduction was observed between 4 to 16 ppm and 64 to 512 ppm, whereas a significant difference (*p* ≤ 0.05) in the reduction was observed between 16 to 64 ppm (Figure 1A). This shows that an optimum concentration of 64 ppm for 30 s to achieve a 5-log reduction of *L. innocua* is required, beyond which increasing the concentration of SHC did not improve log reduction. Further increasing the treatment time up to 5 min increased the log reduction to more than 6 log CFU/mL in the concentration range of 64 to 512 ppm. Below 64 ppm, no difference in the log reduction was observed among the treatment times of 30 s, 1, 2.5, and 5 min (Figure 1A). Similar trends in the reduction were observed for PAA (Figure 1B) and BAC (Figure 1C) treatments of up to 32 ppm. Beyond 32 ppm, a significant increase in the log reduction was observed for both PAA and BAC at 2.5 to 5 min treatment times (Figure 1B,C). Similar findings were reported by Riazi et al. [25] and Belessi et al. [28] when *L. monocytogenes* was subjected to different types of sanitizers. 

To determine strain level variability on log reduction when exposed to sanitizers at different concentrations, two other strains of *L. monocytogenes* (101M, serotype 4b; herein referred as *Lm-1*; and F8385, serotype 1/2b; herein referred as *Lm-2*) were compared with *L. innocua* (non-pathogenic surrogate of *L. monocytogenes*) (Figure 1). A significant *(p ≤* 0.05) difference in reduction was observed among the tested strains. For example, when subjected to 5 min SHC treatment at 32 ppm, *Lm-1* showed a reduction of ≤3.07 log CFU/mL (Figure 1D), identical to the reduction in *L. innocua*, whereas *Lm-2* showed a significantly higher reduction of 5.44 log CFU/mL under the same conditions (Figure 1G). Upon further increasing the sanitizer concentration from 64 to 512 ppm, no significant difference in reduction was observed for *Lm-1* and *Lm-2* (Figure 1D,G). This shows that the reduction trends at low (4 to 16 ppm) and high (64 to 512 ppm) concentration ranges were found to be similar across the tested strains, but the extent of log reductions varied between *L. monocytogenes* strains and *L. innocua* (Figure 1A,D,G). The *L. monocytogenes* strains were found to be more susceptible when compared to *L. innocua,* especially at shorter treatment durations. Similar reduction trends were observed for treatments with PAA (Figure 1E,H) and BAC (Figure 1F,I).

Tamburro et al. [29] demonstrated that different strains of *L. monocytogenes* may have over or under-expression of some genes responsible for resistance. Although concentration of sanitizer(s) was found to be the key factor in the reduction, beyond a certain point, further increasing the concentration had no effect on the tested planktonic cells of *Listeria*. Increasing the treatment time was found to have more effects on log reduction at higher concentrations compared to the lower sanitizer concentrations. Depending upon the concentration and treatment time, the type of sanitizer was found to have a significant effect (*p ≤* 0.05) on log reduction. Under the tested conditions, *L. innocua* was found to be slightly more resilient compared to *L. monocytogenes* strains.

### 3.2. Effect of Re-Exposure to the Same and/or Different Sanitizers 

Based on the findings of sanitizer susceptibility tests as discussed in Section 3.1, *L. innocua* was chosen to report the results of re-exposure tests. The survivors of initial sanitizer(s) exposure at the highest concentration and treatment times (256 ppm for 30 s or 1 min) were first identified, and those cells were again subjected to either the same (co-resistance) or different (cross-resistance) sanitizer treatments at concentration ranges of 64 to 512 ppm. The results were compared with parent strains that were treated for the first time and had not been exposed to sanitizers previously. Table 1 shows the effect of re-exposure to the same or different sanitizer on *L. innocua.* As discussed before, no significant difference in the reduction was observed when the parent strain of *L. innocua* was subjected to 64 to 512 ppm of SHC, BAC, and PAA, respectively. Except in a few instances, treatment time had no significant effect within the tested concentration range. When we compared the susceptibility of parent strains of *L. innocua* exposed to a particular sanitizer with cells previously exposed to the same sanitizer, no significant difference (*p* > 0.05) was found in the case of PAA. BAC treatment of pre-exposed cells showed less reduction compared to treatment of parent cells of *L. innocua* in the treatment times ranging from 1 to 5 min. No consistent trend was observed in the case of SHC treatment between parent and pre-exposed cells (Table 1). This shows that depending upon the type of sanitizer, pre-exposed cells either showed the same susceptibility (PAA) or lower susceptibility (BAC) when compared with their previously unexposed counterparts. In the case of cells that were subjected to different sanitizer treatments after the first exposure to a particular sanitizer (i.e., cross-resistance), BAC pre-exposed cells showed somewhat similar reductions upon re-exposure to SHC, while PAA pre-exposed cells showed higher reductions than parent cells subjected to SHC treatment. Similar trends were observed in case of the BAC treatment of cells pre-exposed to SHC and PAA, whereas *L. innocua* cells that were pre-exposed to PAA showed significantly lower reduction upon re-exposure to SHC or BAC. This indicates that the cells that survived PAA treatment in the first place were able to better resist the exposures to SHC and BAC. However, these differences were in the order of 1 to 1.5 log only at higher sanitizer concentrations (i.e., 512 and 256 ppm), while the difference was as high as 3 log units at lower concentrations of SHC and BAC. Similar trends were mostly observed in the case of other tested strains of *Lm-1* and *Lm-2* (data not shown). 

Fletcher et al. [30] reported that chemical stresses such as exposure to antimicrobial compounds may initiate the overexpression of resistance genes, resulting in decreased susceptibility to the same or other antimicrobial agents. For example, Bland et al. [31] showed that exposure of *Listeria* to 3–4 ppm of quaternary ammonium compound-initiated adaptation to several therapeutic antibiotics such as chloramphenicol, ciprofloxacin, clindamycin, kanamycin, novobiocin, penicillin, and streptomycin. Warth et al. [32] showed that pre-exposure to benzoic acid caused a 1.4- to 2.2-fold increase in MIC of yeasts, and the cellular mechanism is thought to increase cellular efflux, but there is no evidence of such adaptive resistance to benzoic acid in bacteria. A few studies reported resistance development in bacterial cells upon exposure to sanitizers or antimicrobial compounds [10,33], whereas other studies found no association between resistance development and persistence with sanitizer exposure [34,35]. In the present study, previously sanitizer-exposed *L. innocua* showed some level of persistence compared to non-exposed cells when treated with BAC, but no change in persistence was observed when treated with SHC or PAA. Further studies based on an understanding the molecular mechanism of pathogen stress response when exposed to lethal and sublethal concentrations of the same and different sanitizers would be helpful in better understanding their persistence. 

### 3.3. Effect of Repeated Sanitizer Exposure on the Survivability of L. monocytogenes and L. innocua

Table 2 shows the effect of repeated exposure to progressively increasing concentrations of PAA and BAC from 1 to 128 ppm on the survivability of *L. monocytogenes* and *L. innocua*. The results showed that all three tested strains of *Listeria* were able to adapt to BAC and PAA concentrations of up to 32 ppm. Beyond 32 ppm, none of the tested strains were able to grow in BAC. However, the survived cells at 32 ppm of PAA were still able to grow at 64 ppm of PAA. Beyond 64 ppm, further increasing the PAA concentration, no growth was observed (Table 2). This indicates that repeated exposure to sublethal concentrations of sanitizer possibly helped the cells to adapt to higher concentrations that otherwise showed significant reductions when tested on parent cells (Figure 1). Studies reported that the effect of multiple exposures to sublethal concentrations might lead to the development of resistance of that strain against the same or other types of antimicrobial agents [36]. Gao et al. [37] reported that after several exposures to sodium hypochlorite at below MIC, four among nine strains of *Listeria* exhibited resistance. Survivability of cells at sublethal concentration of sanitizers could be attributed to physiological changes in outer cell membrane phospholipid composition or potential genotypic changes at the mar operon [17].

### 3.4. Effect of Sublethal Exposure to Sanitizers on Biofilm Forming Ability of Listeria spp.

**Figure 2:**
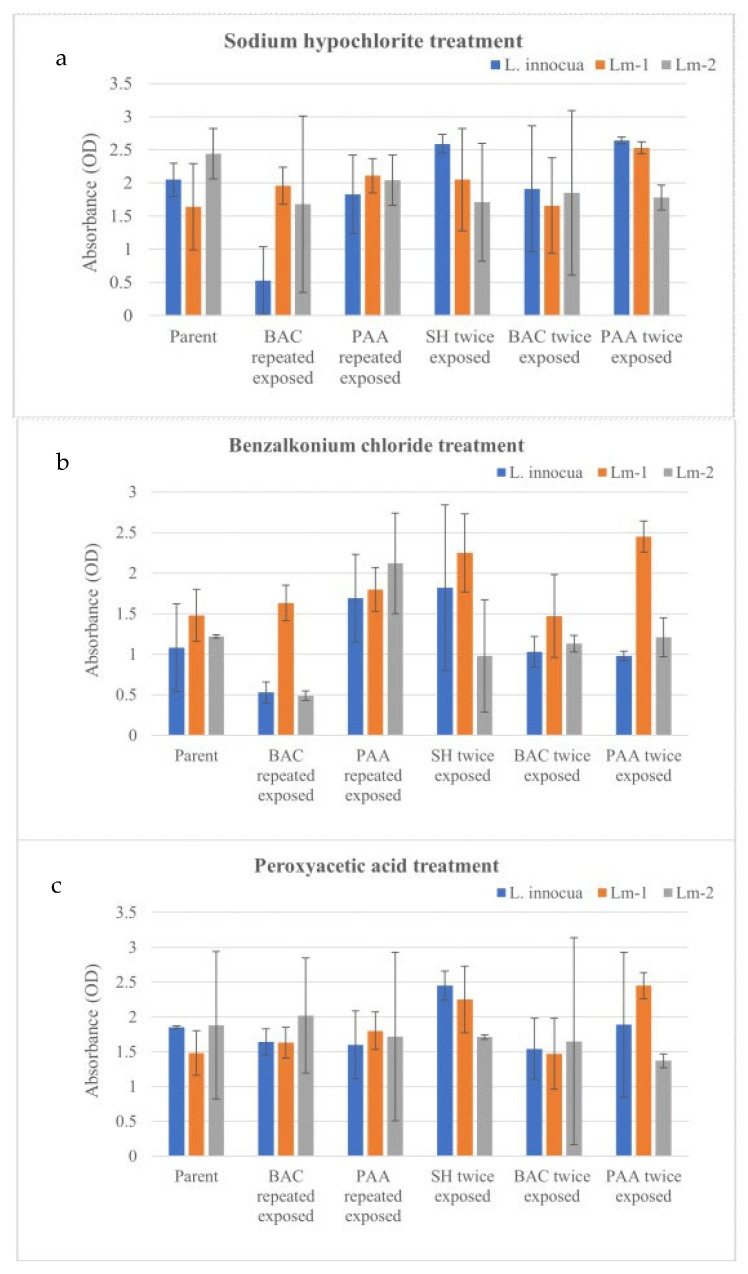
Measurement of biofilm production ability of different *Listeria* strains (which were either pre-exposed or not exposed to different sanitizers) in 16 ppm of (**a**) sodium hypochlorite (SHC), (**b**) benzalkonium chloride (BAC), and (**c**) peroxyacetic acid (PAA) for 72 h.

The effect of prior exposure to sub-lethal concentrations of sanitizers on the biofilm forming ability of *Listeria* was studied. Figure 2 shows the effects of SHC, BAC, and PAA exposure at 32 ppm on the biofilm forming ability of: (i) parent (i.e., cells that had not been exposed to sanitizers before), (ii) cells that survived repeated exposure to sanitizers as described in Section 3.3., and (iii) cells that survived upon dual exposure to sanitizers as described in Section 3.2, respectively. Concentrations of sanitizers in the range of 2 to 16 ppm showed no significant difference (*p* > 0.05) in their effects on biofilm forming ability over a 24, 48, and 72 h period [Supplementary data]. Biofilm production ability after 72 h of exposure to 16 ppm sanitizer concentrations is reported in Figure 2. Treatment with sodium hypochlorite did not elicit a significant difference in biofilm production ability (Figure 2a). Sanitizer-exposed and -unexposed cells showed similar capabilities in biofilm production except for *L. innocua* previously exposed to BAC. Similar findings were observed in the case of PAA treatment of the respective cells (Figure 2c). No significant differences were observed between tested *Listeria* strains as well. When compared to SHC and PAA treatments, BAC exposure resulted in lower levels of biofilm production, especially in the case of cells that survived prior repeated exposure to BAC treatment (Figure 2b). These findings indicate no significant effect on the biofilm forming ability of cells as a result of sublethal exposures to sanitizers. Sanitizer-exposed cells were found to be equally capable of producing biofilm as parent cells, especially under the scenario where they were subjected to sublethal concentrations of sanitizers. Kostaki et al. [38] found that persistent strains had increased biofilm formation or tolerance to stress conditions such as disinfection, but Sharma et al. [39] found no association between persistence and these specific phenotypic characteristics. However, when we compared across the sanitizers, there was a significant difference (*p* < 0.05) in biofilm formation. A study by Luque-Sastre et al. [40] reported that quaternary ammonium compound–based sanitizer was more effective than ethanol-based sanitizers against *L. monocytogenes* and *L. welshimeri* biofilms. To observe irrecoverable damage to the organisms, optimum concentrations of sanitizers at recommended doses are warranted. For example, Hua et al. [41] reported 3.0–3.7, 4.0–4.5, and 2.6–3.8 log 10 CFU/coupon reductions in *L. monocytogenes* biofilms at 400 ppm QAC, 200 ppm PAA, and 200 ppm chlorine concentrations for 5 min, respectively. 

### 3.5. Effect of Sublethal Sanitizer Exposure on Intestinal Cell Interaction with L. innocua 

**Figure 3:**
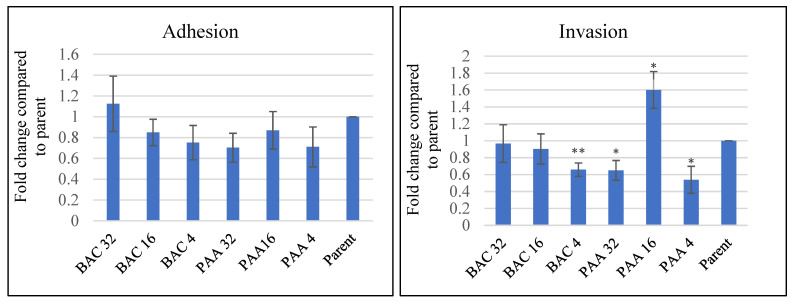
CaCO-2 cell adhesion and invasion of repeatedly sanitizer-exposed *L. innocua* compared to parent strain. BAC 4, 16, 32 and PAA4, 16, 32 refers to *L. innocua* cells that were previously subjected to repeated treatment with peroxyacetic acid and benzalkonium chloride at 4, 16 and 32 ppm, respectively. The adhesion and invasion of *L. innocua* to Caco-2 cells were expressed as a fold change relative to the untreated parent strain. The error bars represent the standard error of the mean. * *p* < 0.05 and ** *p* < 0.01.

The adhesion and invasion ability of repeatedly exposed *L. innocua* in Caco-2 cells were compared with the parent strain. As shown in Figure 3 (Left), both the PAA- and BAC-exposed *L. innocua* exhibited less adherence to Caco-2 cells compared to the parent strain, but the difference was not statistically significant (*p* > 0.05). For invasion, *L. innocua* treated with BAC at 4 ppm showed a significant 0. 65-fold decrease in the invasion of Caco-2 cells compared to the parent strain (*p* = 0.007), while those treated with higher concentrations of BAC did not show significant invasion (Figure 3, right). Interestingly, PAA at 4 and 32 ppm caused a significant decrease (4 ppm: 0.54 fold, *p* = 0.03; 32 ppm: 0.65 fold, *p* = 0.03) in the invasion of Caco-2 cells compared to the parent strain. Surprisingly, repeatedly exposed *L. innocua* treated with PAA at 16 ppm significantly increased the invasion of Caco-2 cells by 1.65 fold (*p* = 0.039) relative to the parent strain (Figure 3, right). One possible reason for the increased invasiveness of *L. innocua* exposed to PAA at 16 ppm may be due to changes in *L. innocua* cell wall structure, which was diminished by higher doses of PAA. Studies using transmission electron microscopic (TEM) analysis have reported that the ultrastructures of the cell envelope and cytoplasm of *L. innocua* were altered after exposure to acidic sanitizers [42]. Such a mechanism might explain how PAA at 16 ppm affected the invasion of *L. innocua*; however, it is still intriguing that repeatedly exposed *L. innocua* treated with PAA at 32 ppm and 4 ppm showed a similar invasion ability. In either case, the mechanism of cell invasiveness is still unclear, and further research is needed to elucidate the detailed mechanism of this phenomenon.

**Figure 4:**
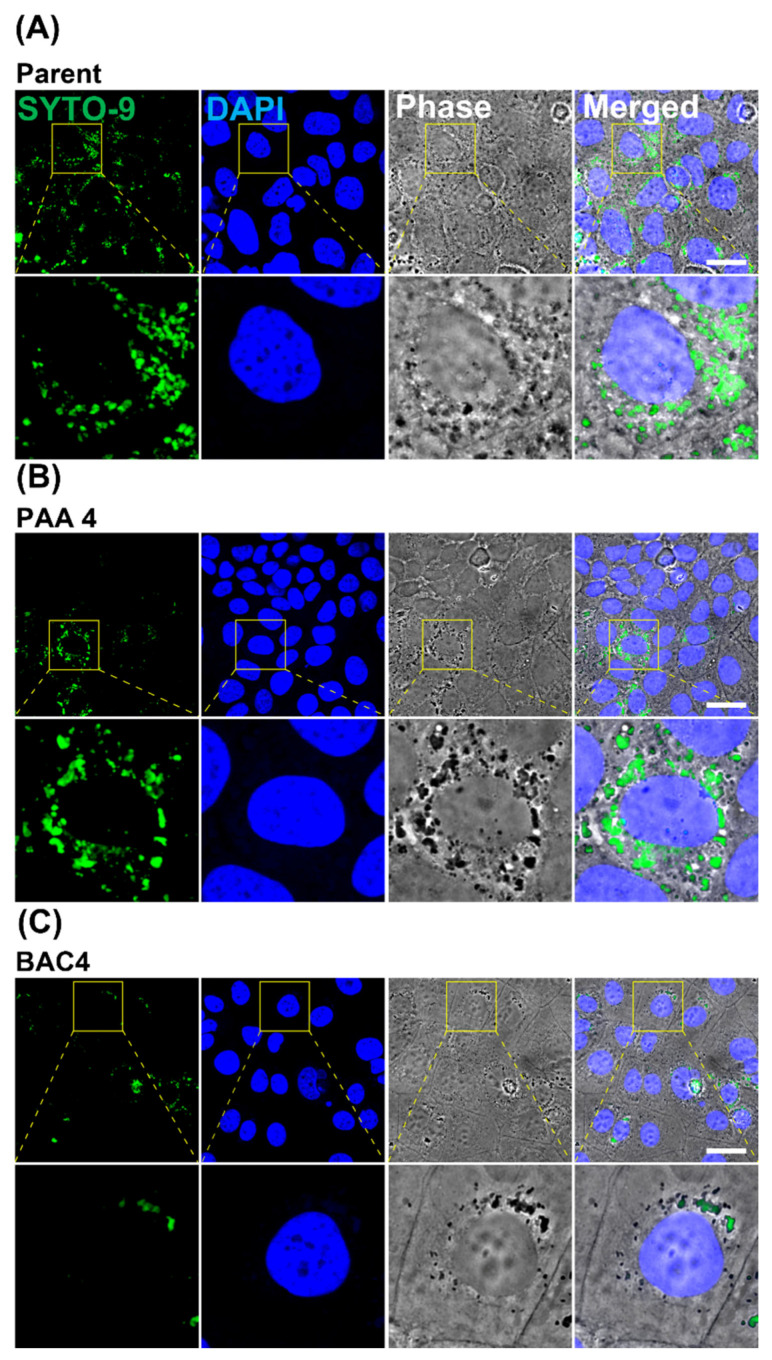
Preexposure of *L. innocua* to PAA or BAC decreased its invasiveness into Caco-2 cells. Representative confocal image showing SYOT-9-stained *L. innocua* bacterial invasion of Caco-2 cells in the parent (**A**) after exposure to PAA (4 ppm) (**B**) and BAC (4 ppm) (**C**). The bottom images in each panel are magnified versions of the square areas depicted in the top images. Scale bar = 30 µm. Green channel: SYTO-9-stained nuclei of *L. innocua* (excitation 485 nm, emission 501 nm). Blue channel: DAPI-stained nuclei of Caco-2 cells (excitation 359 nm, emission 457 nm). Gray channel: phase contrast, merged: the image of SYTO-9 and DAPI staining and phase contrast were overlayed. Experimental details are described in the Materials and Methods section.

To further validate the decrease in invasion of *L. innocua* treated with sublethal concentrations of PAA and BAC, we performed confocal analysis using SYTO 9, a fluorescent nucleic acid stain widely used in food safety analysis to stain live bacteria [43]. Confocal images of Caco-2 cells invaded by *L. innocua* previously exposed to BAC 4 ppm and PAA 4 ppm indicated less SYTO 9-positive *L. innocua* compared to the parent strain (Figure 4). In summary, these data demonstrated that sublethal exposure of *L. innocua* to sanitizers significantly altered invasion while not significantly affecting the adhesion to Caco-2 cells. 

The Caco-2 cell line mimics the intestinal mucosa, thus providing a suitable in vitro tool for investigating the pathogenicity of any bacterium and drug pharmacokinetics [44]. The pathogenicity of *Listeria* originates from its capacity to adhere, invade, and multiply within intestinal mucosa [45]. The ability of *Listeria* to adhere to and invade the Caco-2 cell line varies widely depending on the strain [46]. Ortiz-Sola et al. [47] reported an elevated ability (67.5–81.1%) of *Salmonella enterica* to adhere to Caco-2 cells after disinfection with NaClO (200 ppm). In this present study, no trend was observed that proved the ability of *L. innocua* to adhere to or invade Caco-2 cells was increased or decreased after sublethal exposure to sanitizers.

## 4. Conclusions

The findings of this study showed a reduction of 1 to 8 log CFU/mL across all of the tested sanitizers depending upon treatment time and concentration. As expected, increasing sanitizer concentration and treatment time increased the log reduction, but after reaching an optimum sanitizer concentration, further increasing the concentration of sanitizer did not improve the log reduction. Under the tested conditions, *L. innocua* was found to be slightly more resilient compared to *L. monocytogenes* strains. Sanitizer pre-exposed cells either showed the same reduction (PAA) or lower reduction (BAC) when compared with their previously unexposed counterparts. *L. innocua* cells that were pre-exposed to PAA showed significantly lower reduction upon re-exposure to SHC or BAC. When compared to SHC and PAA treatments, BAC exposure resulted in lower levels of biofilm production, especially in the case of cells that survived prior repeated exposure to BAC treatment. No difference in Caco-2 cell adhesion was observed between sanitizer-exposed and unexposed *L. innocua*. Further studies need to be conducted to fully elucidate the invasion ability of *Listeria* upon sublethal exposure to sanitizers. The findings of this study help to further our understanding of better sanitizer use practices. However, follow-up studies need to be conducted to mimic real-world conditions under the influence of organic matter and associated molecular mechanisms involved in sanitizer tolerance.

## Figures and Tables

**Table 1:** Log reduction of *L. innocua* when re-exposed to the same and/or different sanitizer treatment.

Sanitizer Concentration (ppm)	Treatment Time (min)	Log Reduction (CFU/mL) of *L. innocua* when Exposed to Same or Different Sanitizers either Once (Parent) or Repeatedly (Survived Cells Re-Exposed)											
		Sodium Hypochlorite (SHC) Treatment				Peroxyacetic Acid (PAA) Treatment				Benzalkonium Chloride (BAC) Treatment			
		^1^ Parent	^2^ SHC Exposed	^3^ BAC Exposed	^4^ PAA Exposed	^5^ Parent	^6^ SHC Exposed	^7^ BAC Exposed	^8^ PAA Exposed	^9^ Parent	^10^ SHC Exposed	^11^ BAC Exposed	^12^ PAA Exposed
512	0.5	5.41 ± 1.05 ^aA^	4.32 ± 1.67 ^aA^	4.49 ± 0.35 ^aA^	7.66 ± 0.89 ^bB^	6.45 ± 0.21 ^bB^	5.58 ± 0.21 ^aA^	4.89 ± 0.21 ^aA^	7.66 ± 0.28 ^bB^	4.88 ± 0.5 ^aA^	6.08 ± 0.01 ^bB^	5.08 ± 0.01 ^aA^	7.66 ± 1.04 ^cB^
	1	6.41 ± 0.35 ^bB^	4.93 ± 0.35 ^aA^	5.37 ± 0.35 ^aA^	7.66 ± 0.35 ^cB^	7.65 ± 0.21 ^bB^	5.31 ± 0.21 ^aA^	5.37 ± 0.21 ^aA^	7.66 ± 0.62 ^bB^	7.65 ± 0.01 ^bB^	6.08 ± 0.01 ^aB^	5.22 ± 0.01 ^aA^	7.66 ± 0.23 ^bB^
	2.5	6.80 ± 0.30 ^aB^	6.08 ± 1.1 ^aB^	6.26 ± 0.09 ^aB^	7.66 ± 0.81 ^bB^	7.65 ± 0.21 ^bB^	6.08 ± 0.21 ^aB^	5.56 ± 0.21 ^aA^	7.66 ± 0.28 ^bB^	7.00 ± 0.01 ^bB^	5.73 ± 0.01 ^aA^	5.32 ± 0.01 ^aA^	7.66 ± 0.01 ^bB^
	5	7.65 ± 1.18 ^bB^	6.08 ± 0.35 ^aB^	6.26 ± 0.35 ^aB^	7.66 ± 0.35 ^bB^	7.65 ± 0.21 ^bB^	6.08 ± 0.21 ^aB^	6.26 ± 0.21 ^aB^	7.66 ± 0.21 ^bB^	7.65 ± 0.01 ^bB^	6.08 ± 0.01 ^aB^	5.56 ± 0.00 ^aA^	7.66 ± 0.83 ^bB^
256	0.5	6.10 ± 0.24 ^bB^	-	4.41 ± 1.30 ^aA^	7.66 ± 0.40 ^bB^	5.66 ± 0.21 ^aA^	5.73 ± 0.21 ^aA^	4.46 ± 0.21 ^aA^	-	5.63 ± 0.01 ^aA^	6.08 ± 0.49 ^aB^	-	7.66 ± 0.09 ^bB^
	1	4.75 ± 1.03 ^aA^	-	6.26 ± 0.35 ^bB^	7.66 ± 0.10 ^bB^	6.36 ± 0.21 ^aB^	5.73 ± 0.28 ^aA^	5.46 ± 0.21 ^aA^	-	6.66 ± 0.01 ^aB^	6.08 ± 1.14 ^aB^	-	7.66 ± 0.10 ^bB^
	2.5	5.75 ± 0.33 ^aA^	-	6.26 ± 1.51 ^bB^	7.66 ± 0.68 ^bB^	6.95 ± 2.06 ^aB^	6.08 ± 1.64 ^aB^	5.52 ± 0.71 ^aA^	-	7.65 ± 0.50 ^bB^	6.08 ± 0.86 ^aB^	-	7.66 ± 0.27 ^bB^
	5	7.65 ± 1.15 ^aB^	-	6.26 ± 0.35 ^aB^	7.66 ± 0.16 ^aB^	7.65 ± 0.21 ^bB^	5.25 ± 0.49 ^aA^	6.26 ± 0.28 ^aB^	-	7.65 ± 1.54 ^bB^	6.08 ± 0.76 ^aB^	-	7.66 ± 0.72 ^bB^
128	0.5	5.70 ± 0.03 ^aA^	-	4.45 ± 0.17 ^aA^	7.16 ± 0.14 ^bB^	6.48 ± 2.75 ^bB^	4.37 ± 2.55 ^aA^	4.54 ± 0.62 ^aA^	-	5.14 ± 0.03 ^aA^	5.06 ± 0.46 ^aA^	-	6.66 ± 1.08 ^aB^
	1	5.58 ± 0.01 ^aA^	-	6.26 ± 0.05 ^aB^	7.66 ± 0.19 ^bB^	7.65 ± 2.63 ^bB^	4.32 ± 2.55 ^aA^	4.76 ± 0.28 ^aA^	-	6.05 ± 0.04 ^aB^	5.58 ± 0.16 ^aA^	-	7.66 ± 0.21 ^bB^
	2.5	5.34 ± 0.08 ^aA^	-	6.26 ± 0.78 ^aB^	7.66 ± 0.06 ^bB^	7.65 ± 0.43 ^bB^	4.25 ± 0.87 ^aA^	5.33 ± 1.69 ^aA^	-	7.65 ± 0.14 ^bB^	4.93 ± 0.74 ^aA^	-	7.66 ± 0.21 ^bB^
	5	6.72 ± 0.02 ^aB^	-	6.26 ± 0.15 ^aB^	7.66 ± 0.01 ^bB^	7.65 ± 0.39 ^bB^	4.35 ± 1.41 ^aA^	5.67 ± 1.83 ^aA^	-	7.65 ± 0.08 ^bB^	4.93 ± 0.11 ^aA^	-	7.66 ± 0.21 ^bB^
64	0.5	5.07 ± 0.12 ^aA^	-	4.02 ± 0.04 ^aA^	6.25 ± 0.01 ^aB^	6.34 ± 0.86 ^bB^	3.55 ± 1.15 ^aA^	4.4 ± 0.62 ^aA^	-	5.68 ± 0.18 ^aA^	4.46 ± 0.20 ^aA^	-	5.23 ± 0.21 ^aA^
	1	5.16 ± 0.04 ^aA^	-	5.26 ± 0.21 ^aA^	7.66 ± 0.01 ^bB^	6.8b ± 0.60 ^bB^	3.75 ± 1.17 ^aA^	5.33 ± 0.9 ^aA^	-	4.75 ± 0.02 ^aA^	4.38 ± 0.25 ^aA^	-	6.36 ± 0.21 ^bB^
	2.5	5.23 ± 0.01 ^aA^	-	6.26 ± 0.66 ^aB^	7.66 ± 0.01 ^bB^	7.65 ± 0.95 ^bB^	4.23 ± 0.89 ^aA^	6.26 ± 1.05 ^bB^	-	7.65 ± 0.07 ^bB^	3.43 ± 0.12 ^aA^	-	7.66 ± 0.21 ^bB^
	5	6.01 ± 0.23 ^aB^	-	6.26 ± 1.03 ^aB^	7.66 ± 0.18 ^bB^	7.65 ± 0.77 ^bB^	4.44 ± 0.91 ^aA^	6.26 ± 2.14 ^bB^	-	7.65 ± 0.29 ^bB^	4.88 ± 0.06 ^aA^	-	7.66 ± 0.71 ^bB^

^1, 5, 9^ *L. innocua* that had not been previously exposed to sanitizer treatment; ^2, 6, 10^ *L. innocua* that was previously exposed and survived up to 256 ppm and 30 s SHC treatment; ^3, 7, 11^ *L. innocua* that was previously exposed and survived up to 256 ppm and 1 min PAA treatment; ^4, 8, 12^ *L. innocua* that was previously exposed and survived up to 256 ppm and 30 s BAC treatment. Superscript with same lowercase letters within the same row and sanitizer treatment are not significantly different; superscript with same uppercase letters within the same column and sanitizer treatment are not significantly different.

**Table 2:** Adaptation of *L. innocua* and *L. monocytogenes* after repeated exposure to consecutively higher concentrations of sanitizers.

Type of Sanitizer		Concentration of Sanitizer (ppm)							
	Strains	1	2	4	8	16	32	64	128
BAC	*L. innocua*	+	+	+	+	+	+	−	−
	*Lm-1*	+	+	+	+	+	+	−	−
	*Lm-2*	+	+	+	+	+	+	−	−
PAA	*L. innocua*	+	+	+	+	+	+	+	−
	*Lm-1*	+	+	+	+	+	+	+	−
	*Lm-2*	+	+	+	+	+	+	+	−

‘+’ sign represents growth, and ‘−’ sign represents no growth of *Listeria* spp. at a specific concentration of sanitizer in Muller Hinton broth after 24 h incubation at 37 °C. BAC: benzalkonium chloride; PAA: peroxyacetic acid.

## Supplementary Materials

The following supporting information can be downloaded at: https://www.mdpi.com/article/10.3390/pathogens11090961/s1, Table S1: Biofilm formation of Listeria innocua (L.i) in the presence of Sodium Hypochlorite; Table S2: Biofilm formation of Listeria monocytogenes 101M (L.m-1) in the presence of Sodium Hypochlorite; Table S3: Biofilm formation of Listeria monocytogenes F8385 (L.m-2) in the presence of Sodium Hypochlorite.
