# Supplementary material for: Analysis of Sanitizer Rotation on the Susceptibility, Biofilm Forming Ability and Caco-2 Cell Adhesion and Invasion of Listeria
Source: Pathogens. 2022 Aug 24;11(9):961. doi: 10.3390/pathogens11090961 (PMC9502273; doi:10.3390/pathogens11090961)
Supplement: Supplementary file 1 [file pathogens-11-00961-s001.zip › pathogens-1824360-supplementary.pdf]

Table S1: Biofilm formation of *Listeria innocua* (L.i) in the presence of Sodium Hypochlorite												
**Incubation Time**	**24 hrs**				**48 hrs**				**72 hrs**			
**Concentration**	**2(ppm)**	**4(ppm)**	**8(ppm)**	**16(ppm)**	**2(ppm)**	**4(ppm)**	**8(ppm)**	**16(ppm)**	**2(ppm)**	**4(ppm)**	**8(ppm)**	**16(ppm)**
Parent *L. i*	0.52±0.04^{abA}	0.35±0.04^{aA}	0.5±0.03^{abA}	0.6±0.06^{bA}	1±0.24^{aA}	1.34±0.33^{aB}	0.63±0.29^{aA}	0.86±0.18^{aA}	0.74±0.87^{aA}	2.13±0.33^{aA}	1.77±0.14^{aAB}	2.05±0.25^{aA}
BNZ Multiple exposed *L. i*	0.62±0.07^{aA}	0.2±0.06^{aA}	0.28±0.18^{aA}	0.35±0.21^{aA}	0.53±0.05^{aAB}	0.33±0.32^{aAB}	0.39±0.12^{aA}	0.43±0.08^{aA}	1.39±0.14^{aA}	0.35±1.01^{aA}	0.64±0.48^{aAB}	0.53±0.51^{aA}
**PAA** Multiple exposed *L. i*	0.4±0.41^{aA}	0.21±0.04^{aA}	1.19±1.16^{aA}	0.64±0.48^{aA}	0.95±0.74^{aAB}	0.25±0.1^{aA}	0.42±0.07^{aA}	0.76±0.16^{aA}	1.75±1.2^{aA}	0.46±0.23^{aA}	1.37±0.49^{aA}	1.83±0.59^{aA}
SH Repeated exposed *L. i*	0.54±0.13^{aA}	0.61±0.08^{aA}	0.42±0.18^{aA}	0.73±0.14^{aA}	1.3±0.36^{aAB}	0.58±0.44^{aAB}	1.3±1.38^{aA}	1.15±0.55^{aA}	2.16±0.9^{aA}	1.94±1.16^{aA}	2.65±0.1^{aB}	2.59±0.14^{aA}
BNZ Repeated exposed *L. i*	0.73±0.4^{aA}	0.66±0.07^{aA}	0.59±0.06^{aA}	1.1±0.38^{aA}	1.56±0.46^{aB}	0.9±0.04^{aAB}	1.08±0.3^{aA}	0.74±0.01^{aA}	2.58±0.02^{aA}	1.9±0.44^{aA}	1.97±0.78^{aAB}	1.91±0.95^{aA}
PAA Repeated exposed *L. i*	0.58±0.27^{aA}	1.05±0.76^{aA}	0.64±0.11^{aA}	0.96±0.18^{aA}	1.22±0.14^{aAB}	1.02±0.28^{aAB}	1.44±1.17^{aA}	1.47±1.08^{aA}	2.73±0.08^{aA}	2.64±0.06^{aA}	2.64±0.12^{aB}	2.64±0.05^{aA}
**Biofilm formation of *Listeria innocua* in the presence of Benzalkonium Chloride**												
**Incubation Time**	**24 hrs**				**48 hrs**				**72 hrs**			
**Concentration**	**2(ppm)**	**4(ppm)**	**8(ppm)**	**16(ppm)**	**2(ppm)**	**4(ppm)**	**8(ppm)**	**16(ppm)**	**2(ppm)**	**4(ppm)**	**8(ppm)**	**16(ppm)**
Parent *L. i*	0.81±0.17^{aA}	0.77±0.21^{aB}	0.55±0.47^{aA}	0.37±0.19^{aA}	0.86±0.29^{aAB}	0.82±0.45^{aAB}	0.46±0.09^{aA}	0.41±0.16^{aA}	1.21±1.58^{aA}	0.79±0.46^{aA}	1.66±0.45^{aB}	1.08±0.54^{aA}
BNZ Multiple exposed *L. i*	0.62±0.37^{aA}	0.2±0.02^{aA}	0.28±0.02^{aA}	0.35±0.02^{aA}	0.53±0.41^{aA}	0.33±0.22^{aA}	0.39±0.06^{aA}	0.43±0.03^{aA}	1.39±0.11^{bA}	0.35±0.2^{aA}	0.64±0.13^{aA}	0.53±0.13^{aA}
**PAA** Multiple exposed *L. i*	0.53±0.21^{aA}	0.22±0.09^{aA}	0.28±0.1^{aA}	0.32±0.02^{aA}	0.5±0.25^{aA}	0.19±0.05^{aA}	0.39±0.04^{aA}	0.49±0^{aA}	1.29±0.39^{aA}	0.31±0.14^{aA}	0.43±0.12^{aA}	1.69±0.54^{aA}
SH Repeated exposed *L. i*	0.65±0.05^{aA}	0.49±0^{aAB}	0.46±0.12^{aA}	0.32±0.1^{aA}	0.71±0.38^{aAB}	0.47±0.3^{aAB}	0.42±0.01^{aA}	0.62±0.17^{aA}	0.62±0.31^{aA}	0.55±0.37^{aA}	1.21±0.25^{aAB}	1.82±1.02^{aA}
BNZ Repeated exposed *L. i*	0.81±0.11^{bA}	0.81±0.11^{bB}	0.29±0.04^{aA}	0.26±0.04^{aA}	1.68±0.05^{aB}	1.5±0.21^{aB}	0.76±0.55^{aA}	0.7±0.57^{aA}	1.12±1.06^{aA}	0.72±0.06^{aA}	1.19±0^{aAB}	1.03±0.19^{aA}
PAA Repeated exposed *L. i*	0.76±0.05^{bA}	0.71±0.08^{bB}	0.25±0.05^{aA}	0.26±0.06^{aA}	1.18±0.21^{aAB}	1.22±0.23^{aAB}	0.42±0.07^{aA}	1.51±1.56^{aA}	1.56±1.33^{aA}	0.73±0.15^{aA}	1.19±0.16^{aAB}	0.98±0.06^{aA}

Biofilm formation *Listeria innocua* in the presence of Peroxyacetic Acid

Incubation Time	24 hrs				48 hrs				72 hrs			
**Concentration**	**2(ppm)**	**4(ppm)**	**8(ppm)**	**16(ppm)**	**2(ppm)**	**4(ppm)**	**8(ppm)**	**16(ppm)**	**2(ppm)**	**4(ppm)**	**8(ppm)**	**16(ppm)**
Parent *L. i*	0.66 ± 0.2^{aA}	0.7 ± 0.02^{aA}	0.68 ± 0.07^{aA}	0.67 ± 0.14^{aAB}	0.7 ± 0.05^{aA}	0.73 ± 0.1^{aA}	0.6 ± 0.06^{aA}	0.55 ± 0.14^{aA}	1.12 ± 1.32^{aA}	1.9 ± 0.6^{aA}	1.74 ± 0.35^{aA}	1.85 ± 0.02^{aA}
BNZ Multiple exposed *L. i*	0.45 ± 0.04^{aA}	0.55 ± 0.22^{aA}	0.57 ± 0^{aA}	0.4 ± 0.01^{aA}	0.6 ± 0.04^{bA}	0.4 ± 0.06^{abA}	0.34 ± 0.07^{aA}	0.48 ± 0.04^{abA}	1.93 ± 0.06^{aA}	1.66 ± 0.23^{aA}	1.52 ± 0.28^{aA}	1.64 ± 0.19^{aA}
**PAA** Multiple exposed *L. i*	0.55 ± 0.09^{aA}	0.51 ± 0.12^{aA}	0.47 ± 0.08^{aA}	0.49 ± 0.1^{aAB}	0.49 ± 0.19^{aA}	0.35 ± 0.03^{aA}	0.46 ± 0^{aA}	0.7 ± 0.11^{aA}	1.46 ± 0.12^{aA}	1.57 ± 0.09^{aA}	1.47 ± 0.19^{aA}	1.6 ± 0.49^{aA}
SH Repeated exposed *L. i*	0.56 ± 0.06^{aA}	1.44 ± 1.11^{aA}	1.21 ± 1.03^{aA}	0.61 ± 0.02^{aAB}	0.52 ± 0.28^{aA}	0.82 ± 0.25^{aA}	0.72 ± 0.02^{aA}	0.6 ± 0.06^{aA}	1.94 ± 1.05^{aA}	2.14 ± 0.78^{aA}	2.02 ± 0.69^{aA}	2.45 ± 0.21^{aA}
BNZ Repeated exposed *L. i*	0.66 ± 0^{aA}	0.77 ± 0.15^{aA}	0.94 ± 0.61^{aA}	0.75 ± 0.43^{aAB}	0.95 ± 0.19^{aA}	1.05 ± 0.34^{aA}	1.09 ± 0.34^{aA}	1.02 ± 0.31^{aAB}	1.95 ± 0.09^{aA}	1.74 ± 1.1^{aA}	1.27 ± 0.43^{aA}	1.54 ± 0.44^{aA}
PAA Repeated exposed *L. i*	0.93 ± 0.33^{aA}	0.72 ± 0.45^{aA}	0.7 ± 0.4^{aA}	1.39 ± 0.39^{aB}	2.17 ± 1.44^{aA}	2.29 ± 0.39^{aB}	1.85 ± 1.37^{aA}	2.09 ± 0.68^{aB}	2.06 ± 0.97^{aA}	2.2 ± 0.67^{aA}	1.94 ± 1.06^{aA}	1.89 ± 1.04^{aA}

Table S2: Biofilm formation of *Listeria monocytogenes* 101M (L.m-1) in the presence of Sodium Hypochlorite

Incubation Time	24 hrs				48 hrs				72 hrs			
**Concentration**	**2(ppm)**	**4(ppm)**	**8(ppm)**	**16(ppm)**	**2(ppm)**	**4(ppm)**	**8(ppm)**	**16(ppm)**	**2(ppm)**	**4(ppm)**	**8(ppm)**	**16(ppm)**
Parent *L. m 1*	0.71 ± 0.45^{bB}	0.56 ± 0.06^{aA}	0.65 ± 0.12^{abA}	0.54 ± 0.02^{aA}	1.2 ± 0.27^{bB}	0.57 ± 0.37^{aA}	0.68 ± 0.15^{aA}	0.69 ± 0.36^{a}	0.73 ± 0.86^{aA}	1.31 ± 0.12^{bA}	1.72 ± 0.61^{bA}	1.64 ± 0.65^{bA}
BNZ Multiple exposedL. m 1	0.36 ± 0.28^{aA}	0.45 ± 0.38^{abA}	0.44 ± 0.04^{abA}	0.56 ± 0.21^{bA}	0.36 ± 0.09^{aA}	0.35 ± 0.17^{aA}	0.51 ± 0.19^{aA}	0.54 ± 0.34^{a}	1.16 ± 0.51^{aA}	0.92 ± 0.68^{aA}	1.42 ± 0.72^{abA}	1.96 ± 0.28^{bA}
**PAA** Multiple exposed *L. m 1*	0.33 ± 0.09^{aA}	0.47 ± 0^{aA}	1.16 ± 0.71^{bB}	0.54 ± 0.02^{aA}	1.07 ± 0.36^{bB}	0.34 ± 0.24^{aA}	0.43 ± 0.12^{aA}	0.89 ± 0.4^{6b}	1.43 ± 0.2^{aA}	1.29 ± 0.86^{aA}	1.71 ± 0.26^{abA}	2.11 ± 0.26^{bA}
SH Repeated exposed *L. m 1*	0.44 ± 0.01^{aA}	0.53 ± 0.28^{aA}	0.51 ± 0.08^{aA}	0.57 ± 0.09^{aA}	0.68 ± 0.32^{aA}	0.51 ± 0.28^{aA}	0.47 ± 0.28^{aA}	0.67 ± 0.49^{a}	2.29 ± 0.52^{aB}	1.98 ± 0.87^{aB}	1.9 ± 1.01^{aA}	2.05 ± 0.77^{aA}
BNZ Repeated exposed *L. m 1*	0.54 ± 0.11^{aA}	0.53 ± 0.25^{aA}	0.62 ± 0.15^{aA}	0.45 ± 0.06^{aA}	0.87 ± 0.53^{aAB}	1.01 ± 0.12^{aB}	1.91 ± 0.13^{bB}	1.19 ± 0.04^{ab}	2.37 ± 0.3^{aB}	1.77 ± 0.73^{aB}	2.01 ± 0.58^{aA}	1.66 ± 0.72^{aA}

PAA Repeated exposed *L. m 1*	0.33±0.11^{aA}	0.45±0.2^{aA}	1.2±0.97^{bB}	0.79±0.47^{abA}	1.46±0.93^{aB}	1.29±0.65^{aB}	1.6±0.5^{aB}	1.56±0.81^{a}	2.65±0.01^{aB}	2.37±0.32^{aB}	2.54±0.09^{aA}	2.53±0.09^{aA}
**Biofilm formation of *Listeria monocytogenes 101M (L.m-1)* in the presence of Benzalkonium Chloride**												
**Incubation Time**	**24 hrs**				**48 hrs**				**72 hrs**			
**Concentration**	**2(ppm)**	**4(ppm)**	**8(ppm)**	**16(ppm)**	**2(ppm)**	**4(ppm)**	**8(ppm)**	**16(ppm)**	**2(ppm)**	**4(ppm)**	**8(ppm)**	**16(ppm)**
Parent *L. m 1*	0.84±0.42^{aA}	0.89±0.04^{aA}	0.67±0.03^{aA}	0.76±0.38^{aA}	0.85±0.13^{aA}	0.63±0.08^{aA}	0.65±0.11^{aA}	0.64±0.36^{aA}	0.67±0.46^{aA}	1.37±0.15^{bA}	1.65±0.2^{bA}	1.48±0.32^{bA}
BNZ Multiple exposedL. m 1	0.83±0.59^{aA}	0.49±0.21^{aA}	0.71±0.48^{aA}	0.52±0.34^{aA}	0.63±0.16^{aA}	0.62±0.43^{aA}	0.49±0.01^{aA}	0.56±0.19^{aA}	1.62±0.75^{aB}	1.62±0.05^{aA}	1.56±0.15^{aA}	1.63±0.22^{aA}
**PAA** Multiple exposed *L. m 1*	0.75±0.43^{aA}	0.9±0.25^{aA}	0.97±0.41^{aA}	0.76±0.59^{aA}	0.48±0.09^{aA}	0.57±0.12^{aA}	0.51±0.05^{aA}	0.57±0.1^{aA}	1.19±0.9^{aB}	1.76±0.55^{aA}	1.14±1.33^{aA}	1.8±0.27^{aA}
SH Repeated exposed *L. m 1*	0.66±0.28^{aA}	0.59±0.07^{aA}	0.63±0.08^{aA}	0.53±0.33^{aA}	0.66±0.21^{aA}	0.66±0.21^{aA}	0.81±0.49^{aA}	0.89±0.13^{aA}	1.68±0.62^{aB}	2.45±0.3^{aA}	1.97±0.9^{aA}	2.25±0.48^{aA}
BNZ Repeated exposed *L. m 1*	0.65±0.04^{aA}	0.81±0.27^{aA}	0.83±0.22^{aA}	0.9±0.55^{aA}	1.03±0.29^{aB}	1.02±0.44^{aB}	1.36±0.43^{aA}	1.79±0.72^{aB}	2.01±0.47^{aB}	1.57±0.76^{aA}	1.6±0.03^{aA}	1.47±0.51^{aA}
PAA Repeated exposed *L. m 1*	0.91±0.28^{aA}	0.63±0.07^{aA}	0.7±0.18^{aA}	1.39±1.59^{aB}	1.68±1.49^{aB}	1.27±0.16^{aB}	2.4±1.24^{bB}	1.24±0.26^{aB}	2.09±0.74^{aB}	2.16±0.61^{aA}	2.27±0.41^{aA}	2.45±0.19^{aA}
**Biofilm formation of *Listeria monocytogenes 101M (L.m-1)* in the presence of Peroxyacetic Acid**												
**Incubation Time**	**24 hrs**				**48 hrs**				**72 hrs**			
**Concentration**	**2(ppm)**	**4(ppm)**	**8(ppm)**	**16(ppm)**	**2(ppm)**	**4(ppm)**	**8(ppm)**	**16(ppm)**	**2(ppm)**	**4(ppm)**	**8(ppm)**	**16(ppm)**
Parent *L. m 1*	0.84±0.42^{aA}	0.89±0.04^{aA}	0.67±0.03^{aA}	0.76±0.38^{aA}	0.85±0.13^{aA}	0.63±0.08^{aA}	0.65±0.11^{aA}	0.64±0.36^{aA}	0.67±0.46^{aA}	1.37±0.15^{bA}	1.65±0.2^{bA}	1.48±0.32^{bA}
BNZ Multiple exposedL. m 1	0.83±0.59^{aA}	0.49±0.21^{aA}	0.71±0.48^{aA}	0.52±0.34^{aA}	0.63±0.16^{aA}	0.62±0.43^{aA}	0.49±0.01^{aA}	0.56±0.19^{aA}	1.62±0.75^{aB}	1.62±0.05^{aA}	1.56±0.15^{aA}	1.63±0.22^{aA}
**PAA** Multiple exposed *L. m 1*	0.75±0.43^{aA}	0.9±0.25^{aA}	0.97±0.41^{aA}	0.76±0.59^{aA}	0.48±0.09^{aA}	0.57±0.12^{aA}	0.51±0.05^{aA}	0.57±0.1^{aA}	1.19±0.9^{aB}	1.76±0.55^{aA}	1.14±1.33^{aA}	1.8±0.27^{aA}
SH Repeated exposed *L. m 1*	0.66±0.28^{aA}	0.59±0.07^{aA}	0.63±0.08^{aA}	0.53±0.33^{aA}	0.66±0.21^{aA}	0.66±0.21^{aA}	0.81±0.49^{aA}	0.89±0.13^{aA}	1.68±0.62^{aB}	2.45±0.3^{aA}	1.97±0.9^{aA}	2.25±0.48^{aA}
BNZ Repeated exposed *L. m 1*	0.65±0.04^{aA}	0.81±0.27^{aA}	0.83±0.22^{aA}	0.9±0.55^{aA}	1.03±0.29^{aAB}	1.02±0.44^{aB}	1.36±0.43^{aA}	1.79±0.72^{aB}	2.01±0.47^{aB}	1.57±0.76^{aA}	1.6±0.03^{aA}	1.47±0.51^{aA}
PAA Repeated exposed *L. m 1*	0.91±0.28^{aA}	0.63±0.07^{aA}	0.7±0.18^{aA}	1.39±1.59^{bA}	1.68±1.49^{aB}	1.27±0.16^{aB}	2.4±1.24^{aB}	1.24±0.26^{aB}	2.09±0.74^{aB}	2.16±0.61^{aA}	2.27±0.41^{aA}	2.45±0.19^{aA}

**Table S3: Biofilm formation of *Listeria monocytogenes* F8385 (L.m-2) in the presence of Sodium Hypochlorite**												
**Incubation Time**	**24 hrs**				**48 hrs**				**72 hrs**			
**Concentration**	**2(ppm)**	**4(ppm)**	**8(ppm)**	**16(ppm)**	**2(ppm)**	**4(ppm)**	**8(ppm)**	**16(ppm)**	**2(ppm)**	**4(ppm)**	**8(ppm)**	**16(ppm)**
Parent *L. m 2*	0.7±0.25^{aA}	0.41±0.17^{aA}	0.52±0.12^{aA}	0.4±0.13^{aA}	1.36±1.26^{bB}	0.39±0.03^{aA}	0.41±0.12^{aA}	0.46±0.13^{aA}	1.43±1.78^{aA}	2.5±0.2^{bA}	2.21±0.75^{bA}	2.44±0.38^{bA}
BNZ Multiple exposed L. m 2	0.35±0.17^{aA}	0.39±0.06^{aA}	0.37±0.12^{aA}	0.32±0.05^{aA}	0.2±0.01^{aA}	0.33±0.09^{aA}	0.8±0.74^{aA}	0.63±0^{aA}	1.4±0.46^{aA}	1.82±0.34^{aA}	2.08±0.78^{aA}	1.68±1.33^{aA}
**PAA** Multiple exposed *L. m 2*	0.23±0.01^{aA}	0.25±0.01^{aA}	0.27±0.08^{aA}	0.36±0.14^{aA}	0.57±0.15^{aA}	0.27±0.06^{aA}	0.27±0.13^{aA}	0.57±0.18^{aA}	2.06±0.85^{aA}	1.81±0.95^{aA}	1.4±1.67^{aA}	2.04±0.38^{aA}
SH Repeated exposed *L. m 2*	0.62±0.16^{aA}	0.41±0.04^{aA}	0.44±0.18^{aA}	0.76±0.24^{aA}	0.61±0.01^{aA}	0.64±0.47^{aA}	0.43±0.25^{aA}	0.75±0.12^{aA}	1.97±0.98^{aA}	1.83±0.58^{a}	1.83±0.2^{aA}	1.71±0.89^{aA}
BNZ Repeated exposed *L. m 2*	0.62±0.02^{aA}	1.01±0.61^{aB}	0.73±0.16^{aA}	0.53±0.03^{aA}	0.76±0.74^{aA}	1.15±1.08^{bB}	0.72±0.62^{aA}	1.56±1.33^{bB}	2.54±0.18^{aA}	2.18±0.56^{aA}	2.26±0.58^{aA}	1.85±1.24^{aA}
PAA Repeated exposed *L. m 2*	0.61±0.22^{aA}	0.4±0.24^{aA}	0.3±0^{aA}	0.53±0.09^{aA}	1.8±1.25^{aB}	1.57±1.61^{aB}	1.65±1.44^{aB}	1.78±1.23^{aB}	1.8±0.01^{aA}	1.57±0.09^{aA}	1.65±0.01^{aA}	1.78±0.19^{aA}
**Biofilm formation of *Listeria monocytogenes F8385 (L.m-2)* in the presence of Benzalkonium Chloride**												
**Incubation Time**	**24 hrs**				**48 hrs**				**72 hrs**			
**Concentration**	**2(ppm)**	**4(ppm)**	**8(ppm)**	**16(ppm)**	**2(ppm)**	**4(ppm)**	**8(ppm)**	**16(ppm)**	**2(ppm)**	**4(ppm)**	**8(ppm)**	**16(ppm)**
Parent *L. m 2*	0.59±0.04^{aA}	0.68±0.56^{aA}	0.34±0.01^{aA}	0.36±0.19^{aA}	0.93±0.08^{aA}	0.6±0.38^{aA}	0.49±0.09^{aA}	0.5±0.02^{aA}	0.95±1.14^{aA}	0.77±0.18^{aA}	1.61±0.02^{aB}	1.22±0.02^{aB}
BNZ Multiple exposed L. m 2	0.57±0.19^{aA}	0.45±0.23^{aA}	0.29±0.08^{aA}	0.42±0.13^{aA}	0.56±0.24^{aA}	0.31±0.13^{aA}	0.29±0.03^{aA}	0.4±0.01^{aA}	1.98±0.31^{bB}	0.28±0.11^{aA}	0.4±0.08^{aA}	0.49±0.06^{aA}
**PAA** Multiple exposed *L. m 2*	0.59±0.12^{aA}	0.26±0.04^{aA}	0.33±0.11^{aA}	0.59±0.43^{aA}	0.55±0.21^{aA}	0.79±0.34^{aA}	0.42±0.07^{aA}	0.47±0.09^{aA}	1.23±0.12^{bA}	1.19±1.3^{bB}	0.45±0.04^{aA}	2.12±0.62^{bB}
SH Repeated exposed *L. m 2*	0.76±0.03^{aA}	0.68±0.07^{aA}	0.4±0.02^{aA}	0.47±0.18^{aA}	0.7±0.24^{aA}	0.77±0.36^{aA}	0.44±0.13^{aA}	0.49±0.12^{aA}	1.35±0.96^{bA}	0.59±0.28^{aA}	1.84±0.6^{bB}	0.98±0.69^{abAB}
BNZ Repeated exposed *L. m 2*	0.53±0.02^{aA}	0.61±0.04^{aA}	0.28±0.03^{aA}	0.46±0.19^{aA}	1.7±0.01^{bA}	1.22±0.32^{bB}	0.71±0.22^{aA}	0.59±0^{aA}	2.51±0.01^{bB}	0.71±0.18^{aAB}	1.29±0.08^{abB}	1.13±0.1^{abB}
PAA Repeated exposed *L. m 2*	0.73±0.29^{aA}	0.65±0.29^{aA}	0.33±0.11^{aA}	0.28±0.07^{aA}	1.37±0.14^{bA}	1.14±0.19^{bB}	0.66±0.01^{aA}	1.58±1.11^{bB}	2.18±0.16^{aB}	1.21±0.78^{aB}	1.36±0.02^{aB}	1.21±0.24^{aB}
**Biofilm formation of *Listeria monocytogenes F8385 (L.m-2)* in the presence of Peroxyacetic Acid**												
**Incubation Time**	**24 hrs**				**48 hrs**				**72 hrs**			
**Concentration**	**2(ppm)**	**4(ppm)**	**8(ppm)**	**16(ppm)**	**2(ppm)**	**4(ppm)**	**8(ppm)**	**16(ppm)**	**2(ppm)**	**4(ppm)**	**8(ppm)**	**16(ppm)**
Parent *L. m 2*	0.86±0.14^{aA}	0.82±0.25^{aA}	1.22±0.79^{bB}	0.68±0.19^{aA}	0.62±0.25^{aA}	0.68±0.02^{aA}	0.56±0.02^{aA}	0.7±0.11^{aA}	1.66±1.38^{aA}	2.19±0.54^{aA}	1.88±1.12^{aA}	1.88±1.06^{aA}

BNZ Multiple exposed L. m 2	0.82±0.87^{aA}	0.98±0.76^{aA}	0.42±0.19^{aA}	0.51±0.18^{aA}	0.32±0.07^{aA}	0.58±0.27^{aA}	0.32±0.2^{aA}	0.65±0.08^{aA}	1.35±0.58^{aA}	1.7±0.43^{aA}	1.04±0.07^{aA}	2.02±0.83^{aA}
**PAA** Multiple exposed *L. m 2*	0.48±0.18^{aA}	0.55±0.3^{aA}	0.31±0.1^{aA}	1.14±0.63^{bB}	1.15±0.79^{aB}	2.27±0.36^{bB}	1.02±0.38^{aB}	1.15±0.35^{aB}	1.98±1.04^{aA}	2.27±0.78^{aA}	1.79±0.99^{aA}	1.72±1.21^{aA}
SH Repeated exposed *L. m 2*	1.57±1.15^{bB}	1.13±0.33^{bA}	0.69±0.07^{aA}	0.81±0.48^{aA}	0.61±0.02^{aA}	0.63±0.21^{aA}	1.65±1.33^{bB}	0.83±0.49^{aA}	1.48±0.16^{aA}	2.51±0.42^{aA}	1.32±0.07^{aA}	1.71±0.03^{aA}
BNZ Repeated exposed *L. m 2*	0.54±0.06^{aA}	0.8±0.39^{aA}	0.67±0.1^{aA}	1.37±0.58^{bB}	0.79±0.58^{aA}	0.8±0.31^{aA}	1.01±0.97^{aB}	0.85±0.61^{aA}	2.14±0.61^{aA}	1.38±0.17^{aA}	1.75±1.4^{aA}	1.65±1.49^{aA}
PAA Repeated exposed *L. m 2*	1.52±0.69^{bB}	1.14±0.74^{bA}	0.68±0.27^{aA}	1.07±0.6^{bB}	0.83±0.81^{aA}	0.97±0.82^{aA}	1.65±1.61^{bB}	0.65±0.31^{aA}	1.28±0.06^{aA}	1.27±0.16^{aA}	1.35±0.07^{aA}	1.37±0.1^{aA}
